# Harnessing RSPCA Stakeholder Expertise to Co-Produce a Complex Intervention Addressing Childhood and Adolescent Animal Harm

**DOI:** 10.3390/ani15030347

**Published:** 2025-01-25

**Authors:** Suzanne Lawrie, Claire Blakey, Roxanne Hawkins, Joanne M. Williams

**Affiliations:** 1 Clinical and Health Psychology, School of Health in Social Science, Old Medical School, University of Edinburgh, Teviot Place, Edinburgh EH8 9AG, UK; roxanne.hawkins@ed.ac.uk (R.H.); jo.williams@ed.ac.uk (J.M.W.); 2 RSPCA, Parkside, Chart Way, Horsham RH12 1GY, UK; claire.blakey@rspca.org.uk

**Keywords:** animal harm, animal cruelty, children, adolescents, humane education, intervention, cruelty prevention

## Abstract

Childhood and Adolescent Animal Harm (CAAH) is influenced by biological, psychological and socio-environmental risk factors. Interventions to prevent animal harm among children and young people are essential for fostering empathy and reducing future harm. This study explores the perspectives of RSPCA stakeholders, to co-produce a redeveloped version of ‘Breaking the Chain’, an intervention addressing youth animal harm. Semi-structured interviews were conducted with 16 RSPCA employees across a range of departments and geographical locations in England, UK. Thematic analysis was used to identify key insights on target groups, intervention content, delivery methods, and evaluation strategies. Two primary target groups were identified: universal audiences (primary school-aged children) and high-risk youth. Participants advocated for retaining the core content of the existing intervention while modernising resources to address contemporary risk factors, such as online exposure to animal harm while ensuring accessibility for neurodiverse audiences. Face-to-face delivery was favoured, complemented by digital resources. Evaluation was viewed as critical, with a focus on both short-term outcomes and long-term impact. This study highlights the importance of stakeholder involvement in co-producing effective cruelty prevention interventions. Future steps will involve a coproduction study with children and young people to gather their perspectives on the intervention redesign. This will be followed by a pilot and evaluation of the redesigned intervention, incorporating feedback from both the implementers (e.g., teachers, youth offending teams, RSPCA employees) and the recipients (children and young people).

## Simple Summary

Animal harm during childhood and adolescence can be influenced by various personal and social factors. This study worked with RSPCA staff to update and improve the ‘Breaking the Chain’ programme, which aims to prevent animal harm by young people. Interviews with 16 RSPCA employees suggested the programme target two main groups, primary school children and high-risk youth, with a preference for in-person school sessions supported by online resources. Participants favoured keeping the programme’s core topics of sentience and animal welfare, appropriate behaviours, and triggers and consequences of animal harm, while updating resources to address modern challenges such as online animal harm and peer pressure, and ensuring the content is accessible for children with different learning needs, including neurodiverse children. Evaluating the programme’s short-term and long-term success is key. The next steps include involving young people in the programme’s development and addressing practical challenges like referral systems and programme delivery.

## 1. Introduction

Childhood and Adolescent Animal Harm (CAAH) involves complex behaviours and is influenced by a range of biological, psychological and socio-environmental risk factors. Understanding and intervening in these risk factors is important to prevent animal harm and future harm to people [1]. The ‘Violence Graduation Hypothesis’ posits that childhood animal harm may be a precursor to violence against humans in adulthood [2]; however, it has been criticised for relying primarily on retrospective self-reports. The ‘Deviance Generalisation Hypothesis’ suggests that animal harm co-occurs with other antisocial behaviours like bullying, delinquency, substance misuse [3,4] and school suspensions [5]. There is a need to develop effective interventions for children and adolescents most at risk of animal harm. This paper reports an interview study to co-produce a novel animal welfare education intervention, Breaking the Chain, with animal welfare professionals with lived experience of working with children and young people who have harmed animals.

Childhood animal harm has been defined as “Any act, of commission or omission, where a child negatively impacts an animal’s welfare” [6] (p. 193). This definition will be used in this paper because it acknowledges both intentional and unintentional harm and encompasses a spectrum of severity of harmful behaviours.

### 1.1. Risk Factors for Child and Adolescent Animal Harm

For animal welfare education to be effective it needs to reduce or mitigate risk factors for childhood animal harm. Early research on childhood animal harm identified a range of typologies [7], including explorative, unintentional harm due to curiosity or lack of supervision, most often involving young children; pathological, often linked to psychological issues and more common among older children; and delinquent, commonly seen in adolescents and often associated with antisocial behaviour. Other motivations, like control, retaliation and peer reinforcement, also contribute to children’s harmful behaviour to animals [8,9]. Risk factors underpinning childhood animal harm can be grouped into biological, psychological and socio-environmental risks [6,10,11]. When developing intervention content and determining who would benefit most, it is essential to consider various risk factors, including socio-economic influences like exposure to violence, family dynamics and peer pressure, as well as theoretical frameworks such as the Violence Graduation Hypothesis (VGH). While VGH offers insight into potential pathways of violent behaviour, it has faced criticism (as previously noted), particularly for its reliance on retrospective reports. Therefore, acknowledging the contested nature of VGH is crucial in shaping effective interventions.

Age and sex have been linked with risk of childhood animal harm. McEwan et al. [12] found that cruelty behaviours tend to be more prevalent in early childhood, potentially driven by curiosity or exploratory behaviours. However, as children grow older, particularly during adolescence, animal harm becomes more associated with intentional harm and is often linked to other anti-social behaviours. Ascione [7] observed an increase in animal harm behaviours during adolescence, coinciding with the rise of behaviours like bullying, aggression and delinquency. This difference in intentionality is important to consider in the development of interventions to prevent harm. Males generally attribute less moral worth to animals than females [13] and are more likely to engage in harmful behaviours towards animals [14]. Evidence points to a higher prevalence of harmful behaviours among males at various life stages, including childhood [15], adolescence [16] and adulthood [17]. Additionally, males are more likely to witness harmful acts towards animals [18].

In terms of psychological risks, low levels of knowledge of animal welfare needs and animal sentience contribute to animal harm through acts of omission and commission. There is substantial evidence that children’s and adolescents’ knowledge of animal welfare needs is limited [19,20] and that it can be enhanced through educational interventions [21,22]. This relates especially to unintentional harm caused by lack of knowledge. However, knowledge alone may not lead to positive behaviour when other psychological factors are involved including empathy, attachment and behaviour regulation. Even when aware of the harmful consequences, some young people still choose to engage in animal harm, indicating a need for interventions that address both knowledge and the underlying motivations driving such behaviours.

Empathy is crucial in inhibiting aggression and fostering prosocial behaviour and can be differentiated into three types: cognitive empathy (understanding others’ emotions), affective empathy (feeling others’ emotions), and behavioural empathy (acting to alleviate others’ distress) [23]. A deficit in empathy is often linked to increased aggression, lack of ability to recognise and/or indifference to suffering, and childhood animal harm [24,25,26,27]. There are developmental changes in empathy and also moral concerns for animals, which are higher among children than adolescents [13,28,29,30,31]. Animal welfare interventions can be effective in enhancing some aspects of empathy, especially cognitive empathy [32]. However, low empathy and high callous unemotional traits are risk factors for animal harm and may be difficult to change through educational intervention.

Children with secure emotional attachment styles to key people in their lives typically demonstrate higher empathy and are less likely to harm animals [33]. On the other hand, insecure attachment is associated with lower empathy, poorer behavioural regulation and higher risk for childhood animal harm [34,35]. Children who have experienced trauma or abuse may use pets as sources of attachment over humans [36], even though they are at higher risk of animal harm [37]. This attachment to pets can be both a risk and protective factor for animal harm. While some studies suggest that childhood animal harm may be a precursor to interpersonal violence, as outlined in the VGH [2], it is important to exercise caution to avoid overgeneralising these findings.

Even if a child has an understanding of an animal’s welfare needs, has a positive attitude to animals and is attached to a pet, their behaviour can still be harmful to animals. Self-regulation, the ability to control one’s emotions, thoughts and behaviours, has been linked to childhood aggression and childhood animal harm [38,39]. Deficits in self-regulation are associated with trauma, conduct disorders, bullying and other externalising behaviours, all of which are risk factors for animal harm [10,11,39] Thus, interventions to promote positive behaviour to animals need to consider self-regulation and behavioural control.

The contexts in which children live can also pose socio-environmental risk factors for animal harm. Children who witness animal harm, particularly within their families, may learn and replicate these behaviours. Additionally, peer pressure can significantly influence children, increasing the likelihood of engaging in animal harm. In contrast, children without such exposure or influence were less likely to harm animals [6,40,41,42]. Children and young people are also exposed to animal harm online, and there is increasing use of the internet as a platform for animal harm content [43,44,45]. A recent report by the Social Media Animal Cruelty Coalition (SMACC) [46] identified over 5480 individual videos containing acts of harm, covering a 13-month period, across Facebook, TikTok and YouTube. Authors reported the content was most easily found on YouTube; the platform most widely used by children [47]. Carvalho et al. [44] analysed 411 online productions and reported that 61 of these involved children and young people in acts of animal harm. Exposure to violent content online may normalise animal harm and reduce sensitivity to animal suffering. Children exposed to animal harm may model that behaviour, and also develop attitudes that animal harm is acceptable and that animals are not worthy of moral concern.

### 1.2. Interventions to Prevent Child and Adolescent Animal Harm

Animal Welfare Organisations and US Humane Societies focus substantial time and resources on animal welfare interventions to prevent child and adolescent animal harm. However, there is no agreed intervention model to prevent child and adolescent animal harm and an appreciation that interventions are rarely evaluated [48,49]. A growing number of studies are evaluating interventions to create an evidence base of what works and for whom. Williams et al. [50] evaluated the Scottish SPCA’s Rabbit Rescuers programme for children aged 5 to 7, reporting significant improvements in knowledge of rabbit welfare, understanding of rabbits as sentient beings, and a reduction in children’s acceptance of harmful behaviours towards rabbits. Similarly, the Scottish SPCA Prevention through Education programme (for 7- to 13-year-olds) demonstrated a significant increase in children’s knowledge about animals and beliefs regarding the minds of animals [21]. Furthermore, the Animal Guardians programme, a targeted 1-to-1 education and skills-based intervention for children aged 4 to 12 at risk of harming animals, reported significant improvements in welfare knowledge, behaviour towards animals, and both cognitive and behavioural empathy [32]. These evaluations suggest that animal welfare education programmes are effective in reducing risk factors for harmful behaviours among young people, including low welfare knowledge and negative attitudes. AniCare Child [51] and Children and Animals Together (CAT) are US programmes offering therapy and prosocial skills training to aid the development of empathy, self-control and responsibility towards animals, while involving family members. However, these therapeutic interventions lack formal evaluations to measure their effectiveness.

### 1.3. RSPCA and ‘Breaking the Chain’

‘Breaking the Chain’ (BTC) was developed by the RSPCA in 2010 due to increasing cases of child and adolescent animal harm and calls from professionals working with young people for support. It was developed with guidance from teachers and youth offending professionals for adolescents who had harmed animals or were at risk of harming animals. Breaking the Chain includes activities focused on animal welfare needs, sentience, defining and identifying animal harm, and triggers and consequences of animal harm. The aim of the intervention is to improve understanding of animals, increase empathy and ultimately reduce animal harm offending and re-offending.

Breaking the Chain needs to be refined and modernised to keep pace with changes in young people’s lives. The 2024 annual RSPCA Kindness Index survey [52] revealed that 4 in 10 (43%) 16–17-year-olds had witnessed animal harm online, which is almost double the frequency of the wider adult population (22%). Furthermore, in 2024, of the 24 young people referred to the RSPCA education team for support following harmful behaviour towards animals, 50% were diagnosed with one or more mental health concerns or were neurodivergent. Examples ranged from autism and ADHD through to Pathological Demand Avoidance. Thus, to be effective, Breaking the Chain needs to provide resources that meet the needs of a more neurodiverse range of children and adolescents and to tackle new risk factors such as exposure to animal harm online.

### 1.4. The Present Study

This study represents the first phase of the co-production process aimed at redeveloping Breaking the Chain. During this initial phase, semi-structured interviews with RSPCA stakeholders were conducted to gather insights that will inform the redesign of the intervention’s framework.

The concept of co-production is increasingly utilised in the development of complex health interventions, recognised as vital for enhancing their success and real-world application [53]. Co-production involves the integration of diverse stakeholder perspectives, including those directly affected by the issue. This method allows for the identification of priorities, a better understanding of the problem, and collaborative solutions that increase the relevance and effectiveness of the intervention. Co-production can also reduce over-reliance on theoretical frameworks by incorporating practical and contextual understanding from real-world stakeholders. Engaging RSPCA employees as key stakeholders in the process ensures a balanced approach, blending academic theory with lived experiences [54].

### 1.5. Key Aims

This study aims to explore RSPCA employee perspectives on animal harm and prevention interventions for children and young people, with a specific focus on the redevelopment of Breaking the Chain. The key objectives are the following:

1. Explore employee views and experiences regarding the risk factors associated with child and adolescent animal harm.
2. Gather feedback on the existing Breaking the Chain intervention and identify priorities for its redevelopment, including key goals and ambitions.
3. Identify prime target groups for the intervention to ensure effective and tailored implementation.
4. Determine essential content for the redesign of Breaking the Chain.
5. Explore perspectives on the implementation of the intervention, including aspects of delivery and evaluation.

This research is significant as it integrates the perspectives of a range of RSPCA employees across departments and regions, enhancing the intervention’s feasibility for real-world application [55]. Insights gathered will help establish a clear specification for the RSPCA’s goals in redeveloping Breaking the Chain, whilst engaging employees in the redesign process will hopefully foster interest, awareness and investment in the intervention. Incorporating co-production is critical for developing complex interventions, as it increases the likelihood of their success, sustainability and relevance in practical settings [56]. To the best of our knowledge, no other publications have documented the process of co-producing an animal harm prevention intervention for children and young people, making this study a unique and valuable contribution to the field.

## 2. Materials and Methods

### 2.1. Participants

To ensure a diverse representation of the experience of working in animal harm prevention, a purposive sample of 16 participants, a number deemed adequate for data saturation [57], were recruited. Participants were from seven departments within the RSPCA and across eight geographical locations in England, UK. Inclusion criteria required participants to be RSPCA employees/trustees, at least 18 years of age, living in the UK and fluent in English. We recognise that factors such as age, tenure and role within the organisation might influence responses. To mitigate potential biases and capture a broad range of perspectives, we aimed to include a sample representing various roles, lengths of service and demographic backgrounds. Potential participants were identified by a member of the research team currently employed within the Prevention and Education Department of the RSPCA. A recruitment email containing the participant information sheet was forwarded to the potential participants explaining the purpose of the study and inviting participation. Interested participants signed up to the study via an online survey. Demographic information, including age, gender, time employed with the RSPCA, department and ethnicity, was collected at time of sign-up. All 16 participants identified as white, reflecting the demographic composition of the sampled RSPCA departments. We acknowledge this as a limitation, as it may not fully capture the diversity of perspectives, particularly those influenced by different ethnic backgrounds. Participants fell within the age range of 24–64 years, with the majority (81%) aged between 45 and 64 years. A relatively equal representation of females (*n* = 9, 56%) and males (*n* = 7, 44%) took part. The combined years of RSPCA service among participants totalled 253.5 years, with an average length of employment of 15.8 years per individual. Table 1 shows a full breakdown of sample demographics.

### 2.2. Design and Procedure

This qualitative study used semi-structured interviews to collect in-depth data from RSPCA employees on their experiences and perspectives of child and adolescent animal harm, interventions aimed at reducing and preventing child and adolescent animal harm, and views on the current Breaking the Chain intervention. Ethical approval for this study was granted by the University of Edinburgh Clinical and Health Psychology Ethics Committee (22-23CLPS175).

#### 2.2.1. Interview Schedule

Interview questions were based on the Appreciative Inquiry approach [58]. Appreciative Inquiry, a form of action research with a positive and strengths-based focus, seeks to promote positive change by identifying and enhancing existing strengths and best practice of an organisation or intervention. Appreciative Inquiry is a four-phase approach based on the Discovery, Dream, Design and Delivery phases. Discovery-based open-ended questions were included to encourage discussion of background knowledge and experiences in areas relevant to the study (e.g., animal welfare, childhood animal harm, animal harm prevention interventions, education, etc.) and to identify knowledge levels and opinions of the existing Breaking the Chain intervention. The Dream Phase prompted participants to visualise an ideal animal harm prevention intervention for children and young people and to explore their aspirations and ambitions for the redevelopment of the current Breaking the Chain intervention. The Design Phase focused on the identification of potential resources, skills, partnerships, creative ideas, etc., which may be utilised to aid the delivery of the key ambitions. Finally, the Delivery Phase prompted participants to consider delivery methods and potential challenges in the implementation and sustainability of the redeveloped intervention and offer ways to overcome them. Participants were shown a schematic diagram summarising the core areas, lesson topics, activities and desired outcomes of the existing Breaking the Chain intervention to aid responses relating to content questions. The interview schedule and schematic diagram can be viewed in Supplementary Materials.

#### 2.2.2. Interview Procedure

Following sign-up, participants were contacted by a member of the research team to arrange a suitable interview time. All interviews were conducted in a private setting on Microsoft Teams. The semi-structured interview schedule was followed, allowing for flexibility to explore topics that arose spontaneously. Interviews were carried out over a period of 4 months (April–August 2024) and lasted on average 52 m 31 s. All interviews were audio-recorded, with consent, and transcribed with the aid of the Teams recording and transcription function. Participants had one week following their interview date to retract their data, should they wish to. Following this period, the accuracy of the interview transcripts was verified by the lead researcher and transcripts were anonymised with removal of all identifiable information. Data were stored securely online.

### 2.3. Data Analysis

Interview data were analysed using thematic analysis [59]. Transcripts were initially reviewed by one author to identify preliminary codes capturing key concepts that appeared relevant to the research aims. Preliminary codes were then reviewed, refined and grouped together into broader categories, resulting in a more structured coding framework. Following refinement, themes were identified by clustering related codes that captured significant patterns across the data. Themes were refined and reviewed against transcripts to ensure accurate representation of data. NVivo (14) software was used to assist the coding process and organisation of data. To enhance the reliability of the coding process, two researchers independently coded a subset of the transcript excerpts from a wide range of interviews. Coding was compared to assess consistency.

## 3. Results

**Figure 1:**
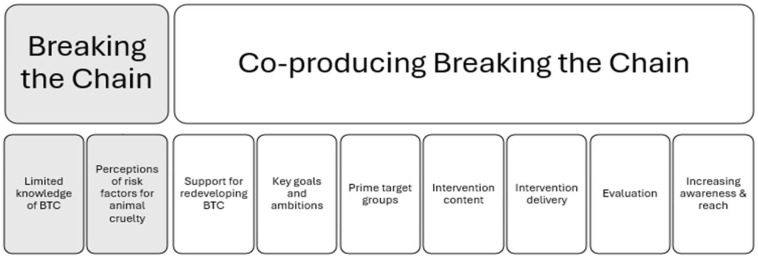
Key themes.

A thematic analysis of interview data identified several key themes central to the redevelopment of Breaking the Chain, as outlined in Figure 1 and detailed below.

### 3.1. Breaking the Chain

#### 3.1.1. Limited Knowledge and Familiarity of ‘Breaking the Chain’ Across RSPCA

While most participants were aware of the intervention’s existence, many participants reported limited knowledge about Breaking the Chain. As one participant noted, “I know the name, and I know roughly what it does” (P13), while another admitted “I don’t know much about it… I would suspect most inspectorate don’t know much about Breaking the Chain” (P11).

Participants generally understood the core aim of Breaking the Chain as preventing animal harm by youth offenders and reducing recidivism, with some referring to it as “an alternative to prosecution” (P13). However, knowledge of the intervention appeared outdated with many participants referring to it in the past tense. One participant reflected, “I knew more about it 10 or more years ago… I know it’s getting on quite a bit now” (P10). None had personally delivered the intervention and most struggled to recall specific details, suggesting awareness and depth of knowledge of the programme is lacking across the organisation.

#### 3.1.2. Perceptions of Risk Factors for Animal Cruelty

Age and Sex: Participants identified a range of risk factors for child and adolescent animal harm focusing mainly on biological factors, particularly age and sex. They repeatedly identified young people within the age range of 10–17 years, with the most common ages referenced being 10/11 and 14/15 years. One participant suggested 15 as “the major pivotal age… where somebody who is just having a lark or just being cruel to animals because their friends are continues or stops it” (P1). Another noted that while, “most serious offences do tend to be the older kids… we do get a fair bit of low-level stuff for younger kids” (P11).

Multiple participants reported that animal harm incidents were more commonly perpetrated by boys. As one participant stated, “a lot of the issues are to do with young males” (P14) and another observed, “It’s definitely more male” (P11). However, P9 noted that although girls may not appear to be involved as often, they are not entirely absent from such behaviour, “it’s not only lads, occasionally girls, but more often lads” (P9).

Social Environments: Environmental factors relating to animal harm included exposure to online harm, peer pressure and culture. Several participants also identified learned behaviour from family members as a significant factor in child and adolescent animal harm.

*“you have families who have poor animal welfare, so they teach their children to be advocates of poor animal welfare”.*

(P12)

Participants highlighted situations where children were seen treating animals in a harsh manner as they had been “exposed to those sorts of behaviours performed by family members” and regarded them “as the norm” (P14). The importance of breaking this cycle and presenting young people with an alternative view on the appropriate treatment of animals was highlighted by P12: “You need to make sure at some point, young people who may be in that bracket are exposed to a different way of thinking about animal welfare”.

Young people demonstrating copycat behaviours of people in the public eye previously exposed for welfare offences were also noted (P9). Participants suggested exposure to online harm is widespread among children and young people, citing data indicating “one in three [or one in four] young people have seen animal cruelty online” (P13, P11). Participants also noted an increase in young people sharing harmful behaviours on social media platforms: “We’re becoming more aware of it, and we’re getting more jobs on it because of the nature of young people having mobile phones, recording it, adding it to… social media platforms” (P5). Instances of harmful behaviours shared online included rats being thrown and hit with a bat, cats being bounced off a bean bag, a puppy being slapped and punched, and birds being kicked. Although the increased visibility of these actions has led to greater awareness, challenges in tracing perpetrators were noted (P9).

Peer pressure was also frequently cited as a driver of animal harm, with children and young people engaging in harmful behaviours to gain social acceptance.

*“it’s that group mentality… that sort of peer pressure… being easily encouraged… and doing things inappropriate but they’re not really giving a huge amount of thought to the potential repercussions of that action”.*

(P5)

One participant described a case in which “a young person ended up playing football with a hedgehog” because of “peer pressure” (P7). Additionally, gang dynamics in inner-city environments were noted as intensifying behaviours with some young people feeling forced to partake in harmful behaviour. As P9 noted, “We have a lot of peer pressure, a lot of gang-related crime involving animals… unfortunately they’ll do things they really don’t wanna do” to prove loyalty to gang members or “keep themselves safe amongst peer groups” (P9).

The cultural context of animal treatment was highlighted as an influential factor, with some communities exhibiting distinct views on animals. One participant noted that a disproportionate amount of time was spent dealing with cases resulting from cultural views: “We deal with them every single day… they take up, percentage wise, a lot more of our time” (P11). These cultural norms and distinct views were seen as being a barrier to interventions, as highlighted by P11: “The trouble with those sorts of families is the parent won’t see there’s a problem… it’s a cultural thing for them, and that’s very hard to break through”.

Psychological and Learning Difficulties: Children and young people with behavioural disorders, learning difficulties, neurodevelopmental differences, mental health challenges or care experience were identified as at higher risk of engaging in animal harm behaviours. According to one participant, cases involving children and young people with “learning disabilities” often involved inappropriate handling, such as “dragging, pulling around, just not handling in what would be considered an appropriate manner” (P5). Emotional and behavioural difficulties, including frustration or anger being directed towards animals, were also described: “They were getting frustrated, frustrated with the dog, frustrated with the parents” (P15), highlighting that some incidents arose from the young person’s struggle with emotional regulation and impulse control. However, it was also noted that acts of harm resulting from behavioural issues “are less numerous” than culture-related incidents (P11).

Lack of support structures and boundaries were also highlighted as contributing factors. P3 discussed the idea that care experienced young people often did not have the necessary skills or support systems to look after animals: “They haven’t had the stability of the family… they’ll go out and purchase an animal for companionship… I don’t think they’ve got the tools to do it correctly… because nobody’s educating them” (P3). This lack of education and support can result in situations where animals suffer due to their young owner’s inexperience, lack of understanding of welfare needs and failure to recognise their behaviours as harmful.

Empathy and Attitudes to Animals: Some young people were described as viewing animals in an objectified manner, treating them as possessions rather than sentient beings. One participant noted, “They saw the dog as a possession” (P7), leading to a lack of empathy and responsibility for the animal’s well-being. Another added, “to think of them as commodities, without personality or anything else, is not conducive to engage empathy” (P16).

Participants also highlighted how young people’s attitudes towards animals varied across species. For instance, one observed, “I wonder… they’re not drawing a distinction between that wild animal and their rabbit or dog at home… they don’t see the hedgehog as a wild animal in quite the same way” (P11), indicating a potential lack of empathy or understanding of wild animals compared to companion animals.

### 3.2. Co-Producing Breaking the Chain

#### 3.2.1. Support for the Re-Development of Breaking the Chain

*“Young people do commit animal welfare crime and some of the worst cases we see are committed by children unfortunately”.*

(P6)

Participants expressed strong support for the redevelopment of Breaking the Chain. All participants rated the importance of this type of intervention as either a four or five (on a scale of 1 to 5), with some stating they would rate it a “six” or “ten” if possible: “It would be a 10… we have to get to the children first and educate them” (P3). Participants viewed it as a proactive approach to prevention, with phrases like “this is what the RSPCA is about, we’re about preventing cruelty” (P10) and “it’s in our title” (P14), emphasising the belief that interventions like Breaking the Chain are fundamental to the organisation’s goals. One participant described the intervention as “vital” suggesting small changes in behaviour could result in “a massive change for so many animals”. While several others stressed the potential long-term benefits of targeting “the future generations” in reducing cruelty and promoting societal change:

*“If we don’t work with people trying to instil these values in them at the start, then we aren’t doing anything in reality to change the future… and these children are the future, they’re the future of the country, they’re the future ambassadors for animals and they’re the future offenders”.*

(P6)

#### 3.2.2. Key Goals and Ambitions for a Re-Developed Breaking the Chain

Key goals and ambitions related to re-developing Breaking the Chain centred around reducing and preventing cruelty through empathy development and changes in attitudes and behaviours.

*“Our biggest aim is to change hearts and minds for all populations, and to treat animals in a much kinder way”.*

(P4)

The long-term reduction in harmful behaviours was discussed as a fundamental goal of the RSPCA’s broader strategy. Several participants referenced the Society’s 2021–2030 Together for Animal Welfare strategy with P11 noting “Ambition One is to reduce cruelty by half… if we’re gonna meet it by 2030… this is where we start”. This sentiment was echoed by P13 who noted “we are looking to reduce animal cruelty and neglect, that’s our big headline figure”.

Prevention also emerged as a key objective. P2 described the need to “prevent the development of an animal harm behaviour pattern in children and young people”, emphasising early intervention to instil positive attitudes and behaviour. P6 further elaborated on this suggesting “it’s all about either preventing that offending happening in the first place, or where it has happened, preventing it happening again”. Participants viewed prevention as twofold: stopping initial harm and preventing recidivism for those who have already harmed animals.

Achieving human behaviour change with the desire to see a clear reduction in animal cruelty incidents was highlighted as a major goal by nine participants (56%). Participants noted the importance of helping individuals “recognize where they’ve gone wrong and what was the right way to do it” (P7 & P12) and in doing so helping change their “attitude, behaviours and responsibility towards animals in the future” and promote an understanding that “every animal has a life worth living” (P12). The development of empathy, respect and broader ethical behaviours was also noted by six participants (38%). P10 explained that fostering greater empathy towards animals might lead to a “knock-on effect” of kindness towards people, broadening the intervention’s impact.

Empowering young people to take action when witnessing animal harm was highlighted by P8 who suggested the need to equip young people with “the tools and strength to be able to report it or call people out”, thus fostering a sense of responsibility and moral action among youth.

#### 3.2.3. Prime Target Groups

Two main target groups were identified for different approaches. Participants stressed the necessity of an intervention targeting high-risk individuals along with a strong desire for universal education to promote empathy and responsibility among all children, potentially reducing cruelty in the long term.

There was widespread support for early intervention, with 11 participants (69%) identifying primary school children as a crucial target group. Many participants advocated for a universal approach beginning in the final year of primary school, when children are better able to rationalise and understand concepts such as responsibility, empathy, and sentience (P4). However, some suggested starting even earlier, with one participant stating, “the younger the better” (P2), while others noted children as young as 5 or 6 are “very receptive to it”, particularly when interactive elements like visits from inspectors were involved (P5, P8).

Participants also supported a multi-stage approach, with repeated interventions as children grow older (P3, P12). As one participant put it, “I don’t believe it’s a one hit wonder” (P3). They suggested revisiting messages during pivotal developmental stages, such as early adolescence (ages 12–14), to reinforce learning. One participant noted RSPCA programmes currently did not do enough to target older children (ages 11–16), particularly teenagers who may be more influenced by peer pressure and social status. This age group was also seen as critical for addressing issues related to gang culture, especially in large cities, and its links to animal harm (P9).

Around one-third of participants emphasised the importance of targeting specific at-risk groups, such as those with a history of animal harm, who they agreed require specialised and individualised interventions. However, these individuals were acknowledged as being among the most difficult to engage (P2). Historical difficulties in identifying at-risk individuals were also noted:

*“I think what the RSPCA has failed to do over the 200 years is… work out who are the people that are likely to be cruel to animals, either deliberately or through lack of education, and how do you get to those tiny percentage of the population”.*

(P1)

Participant 1 highlighted the difficulty in identifying individuals likely to commit animal harm noting the absence of research on which children exhibiting harmful behaviours at a young age will continue these behaviours into adulthood. They recommended focusing on urban areas, which often present more significant challenges due to demographic and socio-economic factors. However, P2 cautioned against assumptions when targeting at-risk young people, stating, “If you’re trying to target children who are at risk, you’re making lots of assumptions”.

#### 3.2.4. Intervention Content

Around half of the participants referred to the core areas of the existing Breaking the Chain (Sentience and Animal Welfare, Appropriate Behaviour, Triggers and Consequences) as “comprehensive” and “really important” aspects of an intervention. There was a consensus all areas were equally important and “mutually interdependent”, with one participant stating, “You can’t separate them… they’re all parts of the same whole” (P16).

Sentience, Empathy and Animal Welfare: Participants consistently highlighted the central role of educating children and young people about animal sentience as a key component of preventing animal harm. Many agreed that this education should begin “at a really young age… ideally very early on in primary school” (P11).

One participant stressed, “it’s not enough just to prevent animals from suffering. We know they’re sentient, so we need to help them to have good lives” (P13). Several participants noted that many children and young people are “not aware these animals have feelings and emotions”. While another described understanding sentience as an “empowering element”, recounting the “light bulb moment” young people experience upon realising animals feel pain and recognising the impact of “what they’ve done” (P7).

Internalising the concept of sentience, rather than simply learning it by rote, was seen as fundamental to developing empathy and understanding of animal welfare (P16, P10). As P14 explained, “If people don’t empathise with animals, then they’re not going to care… you’ve got to have empathy to care”. Sentience was described as a “non-negotiable” aspect of both universal education programmes and targeted interventions (P12).

Participants noted that current frameworks on animal welfare have evolved and should be reflected in the intervention. The inclusion of the “quality of life” concept from The Five Domains Model of animal welfare was highlighted as a relatable way for young people to understand “how the animal feels about its life at that moment in time” and assess the well-being of animals in different contexts such as fighting dogs or farm animals. P14 argued, “Welfare is still quite a technical term… the beauty of talking about quality of life is that people can relate to it very easily”.

Appropriate Behaviours: Participants highlighted the importance of including content on appropriate behaviours within both universal and targeted interventions. P2 described this as a positive approach, explaining that “knowing what the appropriate behaviour is, is very important… it’s positive rather than negative”. Participants stressed the need for children to learn “what is the right and wrong thing to do with animals” (P4) and “what is acceptable” in their treatment of animals (P8). This included handling animals “with respect and compassion” (P4), developing practical skills for safe interactions (P10), and knowing when to refrain from interactions (P16).

Encouraging kindness across species and promoting a broader understanding of animal welfare and humane treatment were seen as key goals. Strategies included challenging harmful social norms, such as dressing dogs in outfits, and addressing misconceptions such as the belief that some animals are of lesser value or suffer less, which leads to the perception that harming them has “not as much of a consequence” (P13). While these elements were considered important, P10 suggested the intervention should prioritise fostering “that connection” between animal sentience and avoiding harmful behaviours, rather than focusing on specific behaviours. They argued that “appropriate behaviour is almost an outcome rather than part of it” (P10).

Some participants raised concerns about using explicit examples of harm in animal welfare education for young children, emphasising the need to tailor content to the age and developmental maturity of the audience (P11). They recommended aligning the intervention with established welfare frameworks, such as the “Five Domains Model”, and focusing on positive guidance to encourage respectful interactions and general kindness towards animals, rather than solely addressing negative behaviours (P14, P2). As P6 explained, “it’s more about… the concept of kindness… our attitude to them rather than about the sort of day-to-day care of them”.

A few participants highlighted the need to expand the intervention beyond just dogs to include “the animals that we have the most incidents with, with young people”, such as companion animals and wildlife (P7). Referring to existing Breaking the Chain content, P3 questioned, “There’s young people and dogs. I don’t understand why it’s just dogs… surely it’s across the board?”

Incorporating content about the behaviour and body language of different species was suggested as a way to help young people better understand these animals. P3 emphasised the importance of teaching ethical treatment for all animals, including farm animals, and reflecting on “how we treat them as a society”. Concerns were also raised about the growing popularity of reptiles as pets and the lack of knowledge surrounding their care (P15).

Triggers for Animal Harm: Several key themes around the importance of understanding and addressing triggers emerged including emotional awareness, challenges with younger audiences and the potential for self-reflection and behaviour change.

*“unless we know the trigger, we’re never gonna do the preventative stuff”.*

(P1)

Many participants emphasised the personal nature of triggers and the role of emotions in impulsive behaviours and triggering harm with P16 noting “in getting to the causes, we’re asking children to examine their emotions”. Some participants felt trigger content was more “appropriate for offenders” (P14) while others questioned the accessibility for younger audiences as understanding triggers requires them to be “emotionally intelligent enough to understand what they’re feeling” (P10). The inclusion of activities, such as emotion cards to “help people connect to their emotions” was proposed as a means of making the content more accessible to younger children. P2 noted further concerns about the risk of “triggering” young people by talking about their emotional triggers, noting that such conversations need to be handled with care.

Although challenges were identified regarding ‘trigger’ content and accessibility to younger audiences, participants highlighted the potential in not only addressing triggers but “dialling up” content (P13). Understanding what triggers harmful behaviours was seen as a gateway to facilitate behaviour change and an important step towards prevention. The reflection process involved in addressing triggers was described as a “penny dropping” moment (P12) in leading perpetrators to see their behaviour for what it is and take accountability for their actions.

Overall, several participants emphasised the importance of emotional education, suggesting that helping young people identify and connect with their emotions should be a focal point of the intervention.

Consequences of Animal Harm: Two-thirds of participants highlighted the significance of addressing the consequences of animal harm within an intervention as a crucial element for deterring harmful behaviours and fostering accountability.

*“I think there’s always a usefulness to make people understand that there are consequences for their actions”.*

(P14)

Several participants stressed the importance of including legal consequences in the intervention, noting that many young people may not be aware of the laws protecting animals (including wild animals) and the “quite significant consequences… which can really impact a young person’s life” (P4). Additionally, P11 noted consequences as a deterrent for dysfunctional, disengaged families suggesting “it is sometimes the consequences that may cause them to step back a bit”.

Some participants proposed the need for consequence-related content to be developmentally appropriate, with more “hard-hitting” content reserved for older children and teenagers while P14 suggested content on consequences may be “appropriate for everyone if it was delivered in a particular way”. P13 advocated for focusing on consequences in a way that is specific to the individual’s behaviour, helping them reflect on their actions and understand the implications. It was suggested restorative justice practices be included within consequences incorporating more information about the “chain of events” following acts of harm, highlighting the long-term impact on both animals and “human victims” and raising awareness amongst young people that animal harm can lead to other criminal behaviours (P13, P16). P2 suggested renaming ‘consequences’ to ‘impact of behaviours’ “might feel a little less punitive”.

Age and Developmental Stage and Culture Appropriate: Participants consistently emphasised the importance of tailoring content to be age- and stage-appropriate, with materials varying in tone and complexity depending on the developmental stage of the audience. For younger children, a positive approach focusing on kindness, compassion and understanding animals’ welfare needs was recommended. P10 reflected, “I wonder whether it’s a question of… doing it in the positive way… this is why we need to be nice to animals”.

For older children and teenagers, participants suggested that more direct and impactful content could be effective. Honest representations of the consequences of animal harm, including physical suffering, were likened to anti-drink-driving campaigns. P9 commented, “I think we need to be a bit more brutal… it’s important we show them the actual pain and suffering”. This approach was seen as aligning with the preferences of teenagers, who, as P11 noted, “don’t want to be mollycoddled”.

A one-size-fits-all approach was widely regarded as unsuitable, particularly in culturally and socially diverse communities where perceptions of animals and animal care may vary significantly. P4 highlighted this challenge: “Our schools and communities are very diverse these days… people have come from areas of the world where animals are treated incredibly differently”. Participants stressed the need for tailored, culturally sensitive interventions, with bespoke approaches adapted to specific demographics. For example, P9 noted the importance of addressing differing challenges and attitudes in inner-city, rural, and affluent areas.

To ensure the intervention remains relevant, participants highlighted the need for regular updates to reflect new research and evolving societal trends such as online animal harm and the influence of peers. As P10 remarked, “Any resources that we produce need to be reviewed regularly” and should be “relatively easy to amend and update as needed”. P7 echoed this, emphasising the importance of a “solid base content” to allow updates without “causing real harm to the resource”. Simplifying the programme to focus on core objectives was also proposed as a way to future-proof the intervention (P10).

#### 3.2.5. Intervention Delivery—The ‘How’

In envisioning how an ideal intervention should be delivered, participants highlighted the importance of face-to-face, small group and one-to-one interventions and a place for online approaches.

In-person versus Digital Intervention: A predominant theme among participants was the perceived superiority of face-to-face interventions, particularly for younger children. Direct human interaction was viewed as more engaging and impactful, enabling better communication and fostering meaningful connections. As P7 observed, interventions tend to be “more successful in person”, while P14 noted that young people “engage so much better when you’re actually standing in front of them”. In-person delivery was also praised for facilitating hands-on learning, such as interacting with live animals or equipment. As P3 explained, “the touch and feel is much better than a screen”. However, participants acknowledged the resource-intensive nature of this approach (P13, P10), which can limit its scalability and reach (P14).

Despite recognising the broader reach of online delivery, participants generally expressed reservations about its effectiveness compared to in-person methods. For example, P11 admitted being “less keen on” digital formats, and P7 described inspiring change online as “a little bit difficult” without the “in-person approach”. Nonetheless, some participants saw value in hybrid models, particularly for reaching older youth and teachers. Participant 6 suggested that “as the children get older, you can probably do more online”, while P10 proposed that online resources could be “attractive to teachers as well”. Digital materials were also seen as a practical solution for overcoming logistical challenges, such as reaching all schools and providing accessible resources for teachers and parents (P3).

Individual versus Group Interventions: Targeted one-to-one interventions were considered essential for high-risk or system-involved individuals. For example, Participant 12 emphasised that “offenders… should have some absolute direct one-to-one” engagement. However, the potentially intimidating nature of a one-to-one setting with one young person and one facilitator was highlighted. Small group work was suggested as an alternative for addressing behaviours occurring in peer contexts. As P2 noted, “young people don’t do this… in isolation, they are usually in a little gang or group”.

Universal prevention programmes were widely supported for group delivery in schools. Integrating animal welfare education into the national curriculum was seen by many participants as the most effective way to reach “as many [young people] as possible”. Schools were identified as a key setting for delivering these interventions in a safe and structured environment, particularly for engaging disadvantaged youth who may lack access to animal welfare education at home (P12). Participant 5 remarked that schools provide an opportunity to “capture all of these children in a safe space”. Additionally, the potential for school-based interventions to foster peer-to-peer learning was highlighted, with P10 suggesting that “getting the message through to [young people]” could result in secondary influence among peers.

Despite the support for school-based interventions, participants also acknowledged challenges. Two participants reflected on past difficulties with the receptiveness of some schools to host interventions (P5, P11). Whilst others recognised difficulties in promoting awareness in schools, noting that proactive communication, such as sending resources directly to schools, or advertising in union magazines may be required to support engagement (P5, P7).

Beyond formal education, three participants advocated for engaging with youth organisations like Scouts, Guides and community centres. These environments were seen as “natural” for fostering discussions about animal welfare (P10). However, it was recognised that these groups, although valuable, may not always reach the most at-risk youth or the “people we’re trying to target” (P9).

#### 3.2.6. Intervention Delivery—The ‘Who’

Participants expressed a strong preference for an integrated approach to intervention delivery, emphasising the need for collaboration between different RSPCA teams. Most participants suggested combining the educational expertise of the Prevention and Education team with practical insights of the Inspectorate to achieve maximum effectiveness. As P14 noted, “an integrated approach” provides “a wider spread and a wider breadth of experience to draw upon”.

Around half of the participants agreed intervention delivery should be “led” by the Education team due to their educational background, delivery skills and experience of working with children and young people. However, many emphasised the value of involving Inspectors playing a “key role” in providing real-world examples that are highly engaging (P11) and can have a lasting impact (P3). P4 noted “there’s always something quite magical for the kids” when inspectors are present in uniform. Potential issues with uniformed staff delivering the intervention were also noted. The perception of RSPCA inspectors as “animal police” may discourage engagement, particularly with adolescents or young people who are less receptive to authority figures (P9, P14).

Many participants advocated for a coordinated multi-departmental approach. While the Prevention and Education team and Inspectorate were most frequently (and equally) referenced, some participants suggested involving other teams, such as Science, Prosecutions, IT, and Press, to ensure the intervention’s factual accuracy (P10). Collaboration with external partners, such as probation officers and social services, who may already be involved with families, was recommended to create a “more joined up approach” (P11, P5, P13).

Lastly, one participant explored the potential of volunteers delivering the intervention. P13 noted a “big growth in volunteering” within the RSPCA and raised the possibility of training volunteers, similar to organisations like the Samaritans. P4 also emphasised the need for consistent delivery across RSPCA branches, noting disparities in school interventions depending on the resources of individual branches.

#### 3.2.7. Awareness of Programme and Enhancing Reach

Participants highlighted the need to raise awareness of the programme, both internally within the RSPCA and externally with key stakeholders. Internally, some participants noted the key role Inspectors could play in identifying and referring young people to the programme provided they were adequately informed about its existence and purpose. As P13 stated, “There’s a job to do around raising awareness that this programme exists” (P13).

Externally, there was a call for multi-agency collaboration to strengthen impact and sustain the programme. As P9 remarked, “Everybody has to be on board with it… stakeholders, partnerships, everyone in animal welfare”. Effective partnerships with schools, local authorities, police forces and other animal welfare organisations were seen as crucial to both delivering the programme and distributing the costs associated with preventing animal harm (P9). Social media was identified as a vital tool for raising awareness. P6 described it as “the most powerful tool” for disseminating information, while P11 stressed, “We need to start getting this stuff out on social media”. Participants advocated for the use of diverse communication strategies, including short films and online campaigns, to showcase the programme’s impact, particularly targeting key referrers such as schools, police forces and youth offending teams (P2, P6). Inspectors were seen as willing contributors to media content aimed at supporting the programme. However, participants also highlighted the importance of selecting trusted messengers to deliver key messages effectively, ensuring resonance with the target audience (P9). One participant underscored the need to publicise the programme: “Schools don’t know about it… the public don’t know about it… we’ve just rebranded, we’re very active on advertising, we’re very active on TV, we’re very active on social media… now’s the time to tell people” (P6).

### 3.3. Evaluation

Participants emphasised the need for both short- and long-term evaluation methods, incorporating multi-stakeholder feedback to comprehensively assess the intervention’s impact. Some suggested using questionnaires or surveys to measure immediate changes in welfare understanding or attitudes towards animals before and after the intervention. However, concerns around children and young people providing socially desirable answers cast doubt on the reliability of such measures (P2).

Qualitative feedback from children and young people in the form of self-reported evaluation stories was noted as another way of capturing short-term behaviour or attitude changes, as were case studies. A reduction in repeated complaints or visits to the same household could be used as an immediate measure of effectiveness. As one participant noted, “In the short term… we can monitor whether or not we get any repeat visits or complaints to the same location involving the same children”. Participants also highlighted the importance of incorporating feedback from various stakeholders including referral sources (e.g., police, social workers, teachers) and the facilitators delivering the programme.

Around one-third of participants noted the importance of evaluating the long-term impact of the intervention. As one participant put it, “Obviously we’re looking at the long-standing outcomes” (P4). They suggested long-term success could be measured by tracking reductions in investigations or complaints of animal harm involving children and young people over time (P4, P5). However, several participants acknowledged the challenges of measuring these outcomes, noting that tangible changes, such as reduced complaints or incidents, might take years to become evident (P5, P6). Ongoing engagement with children and young people and their parents (if appropriate) was also suggested as important for tracking change and reinforcing the intervention’s impact (P15).

Three participants emphasised the importance of demonstrating the intervention’s overall impact on long-term sustainability. As one participant noted, “The proof is in the pudding, isn’t it?” (P11). Another highlighted how creating an evidence base of “changed attitudes, changed behaviours… will give it the credibility it needs to continue” (P13). Sharing best practices where the intervention has been successful was also noted as a critical step for promoting sustainability (P4).

## 4. Discussion

This study represents the initial phase in the co-production process for redeveloping the RPSCA ‘Breaking the Chain’ intervention, aimed at reducing and preventing animal harm among children and young people. To our knowledge, this is the first study to document the co-production of an animal harm prevention intervention for this demographic. Engaging RSPCA employees provided valuable insights into the strengths of the existing intervention and key areas for development. These findings can help ensure the intervention adapts to emerging social, behavioural, cultural and technological shifts in childhood animal harm, address the complex risk factors underpinning harmful behaviours, and in doing so, effectively meet the diverse needs of today’s youth.

### 4.1. Key Findings

A recurring theme was the limited awareness and knowledge of ‘Breaking the Chain’ among RSPCA employees, particularly regarding its content and delivery. Enhancing internal communication, especially within the Inspectorate, and implementing targeted training sessions focused on the intervention’s specifics (such as goals, target audiences, content, delivery methods, evaluation processes and referral pathways) could help to address these knowledge gaps. Improved employee awareness and engagement may enhance the identification and referral of at-risk youth, thereby strengthening the intervention’s reach and efficacy.

#### 4.1.1. Areas of Agreement in Intervention Focus

This study confirmed the intervention’s relevance but highlighted the need to modernise content to address evolving risk factors (e.g., social, cultural and technological influences) of childhood animal harm and the diverse needs of today’s youth. There was strong support for BTC as a proactive approach to fostering empathy and kindness towards animals in future generations, aligning with the RSPCA’s broader mission and strategic goals, and creating meaningful, long-term reductions in harmful behaviours.

Target Groups: Two distinct target groups emerged as priorities: universal childhood audiences (e.g., primary school-aged children) and high-risk youth. Universal interventions were seen as crucial for promoting an understanding of animal sentience and welfare and for fostering empathy with animals early in life. Participants also advocated for targeted interventions tailored to high-risk youth, recognising the complexity of their needs, including exposure to animal harm or maladaptive peer influences. These findings align with previous research highlighting at-risk youth and school-aged young people as the main target groups for such interventions [48]. However, as participants noted, engaging adolescents in such interventions presents unique challenges. Older youth may require context-specific approaches, highlighting the need for tailored strategies. Participants’ emphasis on developing resources that resonate with adolescents provides an opportunity to address this identified gap, as most existing animal welfare interventions disproportionately target younger children [48].

Core Content Areas: The retention and modernisation of the existing intervention’s core content—sentience and animal welfare knowledge, appropriate behaviours, triggers and consequences of animal harm—was widely supported. Additionally, participants advocated for the inclusion of new themes addressing emerging risk factors such as online harm.

Understanding animal sentience and animal welfare was viewed as foundational for fostering empathy and respectful attitudes towards animals. Empathy, a key factor in reducing aggression and encouraging prosocial behaviour [23], was identified as crucial for long-term behaviour change. This aligns with prior research indicating that empathy-focused interventions can reduce aggressive behaviours and support meaningful change [32].

Updating the intervention to reflect current frameworks on animal welfare was also identified as a priority. Evidence suggests that children and young people’s knowledge of animal welfare needs is limited [19,20]. However, this knowledge gap can be effectively addressed through targeted educational interventions, as highlighted in previous studies [21,22].

Practical guidance on respectful interactions with animals was deemed necessary, including content that challenges speciesism and confronts the normalisation of online animal harm and social norms, such as dressing animals inappropriately for human entertainment. Educating young people to recognise and adopt respectful and responsible behaviours towards animals was seen as a strategy to foster healthier human–animal interactions.

To address the root causes of animal harm, participants recommended content on emotional regulation, self-awareness and self-reflection. Content that helps young people identify and manage their emotional responses was seen as essential for reducing impulsive, harmful actions towards animals. Deficits in self-regulation have been linked to childhood aggression and childhood animal harm [38,39] underscoring the importance of incorporating self-regulation activities into the intervention.

Participants also highlighted the value of addressing the legal, emotional and restorative justice implications of cruelty. Developmentally appropriate content tailored to different age groups was seen as crucial for deterring harmful behaviours and fostering accountability.

The need for the redeveloped Breaking the Chain to address modern challenges was emphasised. Integrating content on digital responsibility and ethical social media use was considered essential for equipping young people with skills to identify and respond to witnessing online animal harm. Recent studies, such as Carvalho et al. [44], highlight the increasing role of technology in perpetuating and monetising animal harm, further emphasising the need for digital literacy components within the intervention. To ensure the intervention remains relevant, there is a need for culturally sensitive and neurodiverse resources to recognise the diverse needs of today’s youth.

Delivery Preferences—Modes and Locations: Participants expressed a strong preference for face-to-face interventions, particularly for younger children, suggesting direct engagement may foster deeper, more impactful learning and behaviour change. There was a consensus that schools may be the most effective setting for universal delivery, providing access to broad and diverse audiences in a safe and structured environment [21]. However, acknowledging logistical challenges with school engagement and delivery and the resource-intensive nature of in-person delivery, participants suggested complementary online resources, particularly for older youth and teachers. A hybrid delivery model could combine the engagement benefits of in-person sessions with the scalability of digital tools and the learning styles of today’s youth.

There was a focus on in-house delivery and support for multi-departmental collaboration, with the RSPCA’s Prevention and Education team and the Inspectorate playing key roles. Inspectors were seen as impactful figures due to their real-world experience while Prevention and Education staff have skills in working with young people, resource development and intervention delivery. However, the potential to include collaborations with volunteers, schools and external agencies could be an area for consideration. Although challenges exist regarding collaboration with other animal welfare charities due to the need to maintain distinct interventions, there are ongoing efforts to foster collaboration where feasible. The Pet Education Partnership (PEP) serves as a notable example of such efforts. This partnership, involving eight leading animal welfare organisations in the UK, aims to provide accessible animal welfare education for children aged 5 to 11, with the long-term objective of integrating it into the national curriculum. This type of collaboration enables charities to work together on addressing broader educational goals while maintaining their unique contributions, such as Breaking the Chain.

Evaluation Needs: Robust evaluation was viewed as critical to demonstrating Breaking the Chain’s effectiveness and ensuring its sustainability. Short-term measures such as pre- and post-intervention surveys measuring changes in knowledge, attitudes and behaviours were suggested for providing immediate feedback, whilst qualitative feedback such as case studies was recommended to complement these measures and tackle concerns around young people providing socially desirable answers. Participants acknowledged the challenges of collecting long-term data [48] but highlighted the importance of tracking recidivism rates and cruelty complaints involving young people over time, in assessing the intervention’s impact and potential for long-term behaviour change. Incorporating multi-stakeholder feedback was suggested as a way of further enhancing understanding of the intervention’s effectiveness.

#### 4.1.2. Implementation Considerations

Referral System: The limited discussion around the existing referral process for Breaking the Chain may indicate a lack of awareness among stakeholders. The absence of a clearly defined referral pathway poses a risk of underutilising the intervention for those who would benefit most. Developing a referral protocol that enables RSPCA Inspectors, schools, probation services, local authorities or social services to identify and direct at-risk youth and perpetrators of animal harm towards Breaking the Chain could enhance the provision of timely and targeted support. A comprehensive referral database would also aid long-term evaluation in tracking changes in referral numbers and types of complaints.

Delivery Plan: While participants offered valuable suggestions regarding delivery modes and locations, a comprehensive delivery framework is essential to ensure consistent implementation. Key considerations include the training of facilitators in intervention delivery, child protection and safeguarding, along with clear guidance on where and how interventions, particularly specialised and targeted ones, will be conducted. A manualised approach would aid consistency in delivery. Facilitators must also undergo disclosure checks (e.g., PVG/DBS clearance), with their well-being prioritised through access to emotional support services such as the RSPCA’s TRiM (Trauma Risk Management) and EAP (Employee Assistance Programme) services.

Evaluation Roles and Methodologies: Robust evaluation encompassing both short- and long-term outcomes and impact was widely supported; however, further consideration needs to be given to exploring methods for long-term evaluation and defining evaluation roles and responsibilities within the organisation, including who will conduct evaluations, analyse data and share findings. Delivering targeted interventions in resource-constrained or culturally diverse settings requires careful planning. Tailored approaches, culturally sensitive materials, and partnerships with local organisations could enhance reach and impact. Digital tools could also support scalability and consistency across regions.

### 4.2. Strengths, Limitations and Future Directions

This study used rigorous qualitative interview methods to gain perspectives on the re-design of Breaking the Chain. A strength of the study was the inclusion of a range of participants across various RSPCA departments and geographical locations, providing a broad organisational perspective. While efforts were made to include participants from a geographically diverse group, encompassing both urban and rural areas and regions with differing socio-economic statuses, the lack of ethnic diversity among participants is a notable limitation. This limitation results in an incomplete understanding of cultural and social differences in attitudes towards animal welfare and education. Future research will seek to include a more ethnically diverse sample to enrich the understanding of how these factors influence perceptions and attitudes. Furthermore, the study relied solely on RSPCA employees, excluding perspectives from external stakeholders such as educators, probation officers and social workers. Incorporating a wider range of viewpoints could provide a more comprehensive understanding of the intervention’s potential impact and implementation challenges. Limited familiarity with the existing Breaking the Chain intervention among participants may have constrained the depth of feedback on specific intervention components. Indicating a need for increased internal communication about the intervention.

Future directions include engaging in co-production work with potential recipients of the programme. At the core of co-production is the involvement of individuals who typically lack a voice in the decision-making process, such as children and young people [60]. A crucial next step in redeveloping Breaking the Chain is engaging children and young people, particularly those with lived experience of animal harm, to contribute to the design of content, delivery formats and impactful messaging. As experts in their own lives, their input can significantly enhance the intervention’s relevance, effectiveness and acceptance. Moreover, this approach aligns with the legal and ethical obligation to involve young people in decisions about matters that directly affect them [61]. This participatory approach could pave the way for a more impactful and sustainable intervention. Engaging those with lived experience can ensure the programme resonates with its intended audience and aligns with their needs and preferences [62].

## 5. Conclusions

Participants expressed strong support for the redevelopment of Breaking the Chain, recognising it as integral to the RSPCA’s mission and its 2021–2030 ‘Together for Animal Welfare’ strategy. The findings highlight the intervention’s potential to drive meaningful change by reducing risk factors for animal harm and fostering a culture of empathy and respect for all animals among future generations. With thoughtful redevelopment, Breaking the Chain can play a pivotal role in achieving the RSPCA’s goal of reducing animal harm by 50% in England and Wales by 2030, and their vision of creating a world where all animals are respected and treated with kindness and compassion.

## Figures and Tables

**Table 1:** Demographics of interview participants, *n* = 16.

Variable	Frequency	%
Age		
24–34	1	6.3
35–44	1	6.3
45–54	7	43.8
55–64	6	37.5
Prefer not to say	1	6.3
Gender		
Female	9	56.3
Male	7	43.8
Department		
Companion Animals	1	6.3
Inspectorate	7	43.8
Policy, Prevention and Campaigns	1	6.3
Prevention and Education	2	12.5
Prosecutions	2	12.5
Public Affairs and Campaigns	2	12.5
Trustee	1	6.3
Years with RSPCA		
0–5	2	12.5
6–10	3	18.8
11–15	2	12.5
16–20	4	25.0
20+	5	31.3
Geographical location		
London	3	18.8
Midlands	2	12.5
North East England	1	6.3
North England	2	12.5
North West England	2	12.5
South East England	4	25.0
South England	1	6.3
South West England	1	6.3

## Data Availability

Data can be accessed by contacting the lead author.

## Supplementary Materials

The following supporting information can be downloaded at: https://www.mdpi.com/article/10.3390/ani15030347/s1, Materials 1: Breaking the Chain Summary Schematic Diagram; Materials 2: Interview Schedule.
