# Supplementary material for: Harnessing RSPCA Stakeholder Expertise to Co-Produce a Complex Intervention Addressing Childhood and Adolescent Animal Harm
Source: Animals (Basel). 2025 Jan 25;15(3):347. doi: 10.3390/ani15030347 (PMC11816218; doi:10.3390/ani15030347)
Supplement: Supplementary file 1 [file animals-15-00347-s001.zip › Interview Schedule.pdf]

THE UNIVERSITY of EDINBURGH

RSPCA

Project Title: Harnessing RSPCA Stakeholder Expertise to Co-Produce a Complex Intervention Addressing Childhood and Adolescent Animal Harm

<u>Pre-interview (Approx. 5 min)</u>

- Welcome and introduction to study

<u>Interview Questions (approx. 30 – 50 mins)</u>

<u>Section 1: RSPCA & Animal Cruelty</u>

1. Please tell me a little about any experiences prior to your employment with RSPCA relevant to animal welfare/animal cruelty, education or working with young people.
2. Please tell me a little about your background/experiences within the RSPCA.
3. Have you been involved in any work specifically targeting the prevention of animal cruelty? *If yes, please tell me a little about it.*
   3a) within the RSPCA
   3b) out with the RSPCA
4. How do you envision an animal cruelty prevention intervention for young people to look in its ideal form?
   <u>Prompts:</u>
   - *Who would be the prime **target group**? (and why this group)*
   - *What **content** do you feel would be important in an effective intervention? (do you think this would differ depending on child age, and severity of cruelty?)*
   - ***Who** would be best placed (within the RSPCA) to **deliver** animal cruelty intervention programmes? (Should this be the responsibility of one team or integrated approach)*
   - ***What** do you believe would be the most effective **way to deliver** such an intervention? (e.g., online, in schools, 1-2-1 etc)*
   - ***Evaluation –** what do you think would be the best way to evaluate the impact / success of such program/intervention?*
5. How important do you believe this type of intervention should be to the RSPCA? If you could rate it on a scale of 1 – 5, with 1 being not important at all to 5 being extremely important, what would you say? And Why?
6. In your opinion, what unique strengths/resources does the RSPCA have that could be utilized in developing, delivering, and sustaining an effective animal cruelty prevention intervention?
7. How could current partnerships with key stakeholders enhance the effectiveness of such an intervention?
8. Are you aware of existing programmes, initiatives, or campaigns within the RSPCA that have shown promise in reducing animal cruelty? *If yes, please tell me about them.*
   - *Who are the target audiences?*
   - *Who delivers them and how?*
   - *Can you share any success stories/positive experiences regarding these programmes?*
9. Do you know of any existing programmes, initiatives, interventions out with the RSPCA that aim to prevent / reduce animal cruelty within the UK? *If yes, what are they? Are you aware of any positive impact?*

Section 2: Breaking the Chain (Use resource summary schematic from Q3 for participants unfamiliar with Breaking the Chain)

1. Turning our focus to the RSPCA Education Team, what is your understanding of their role within the organization?
2. Have you heard of the RSPCA Breaking the Chain intervention?
   - *If yes, can you tell me what you know about the intervention? (e.g., what components are you familiar with?)*
   - *Are you able to share any positive experiences or success stories related to the current intervention?*
3. Who do you see as the target group for this intervention? (Are there any specific demographics or communities you believe should be prioritized?)
4. What aspects of the intervention do you feel to be of the most value? Why? (What aspects are most likely to contribute to future success in tackling childhood animal harm? Why?)
5. In your opinion, what aspects of the current intervention align with RSPCA's 2021 – 2030 Strategy: Together for Animal Welfare? (Vision, mission, beliefs, values, ambitions, and priorities)
6. Do you believe this is an intervention the RSPCA should be committed to funding the redevelopment of? *If yes or no, why?*

Section 3: Redeveloping Breaking the Chain

Thinking back to your ideal vision of an animal cruelty prevention intervention:

1. Can you identify any resources/content that are lacking or need improvement from the current intervention to achieve this vision?
2. Can you identify any specific expertise/resources within your department that can be leveraged to help achieve this vision?
3. In your view, what are the key goals/ambitions the RSPCA would be aiming to achieve through the redevelopment of the Breaking the Chain intervention?
4. How can we increase public awareness and engagement with the redeveloped Breaking the Chain intervention?
5. What steps can we take to ensure the sustainability of the intervention for the future? (What do you think would be needed for long term sustainability/what could the barriers to this be?)

Closing questions

1. The next step in the redevelopment process may involve setting up a co-production team to be involved in key decisions around the design of the intervention. Are you happy to be contacted in the future should we require your support as part of the co-production team?
2. Is there anything else you would like to add or talk about that we haven't covered?

Thank you very much for taking part in this interview.

End of interview (approx. 5 mins)

- De-brief and signpost to support service
