# Supplementary figures and images for: Harnessing RSPCA Stakeholder Expertise to Co-Produce a Complex Intervention Addressing Childhood and Adolescent Animal Harm
Source: Animals (Basel). 2025 Jan 25;15(3):347. doi: 10.3390/ani15030347 (PMC11816218; doi:10.3390/ani15030347)

## BREAKING THE CHAIN – PROGRAMME/RESOURCE SUMMARY DIAGRAM

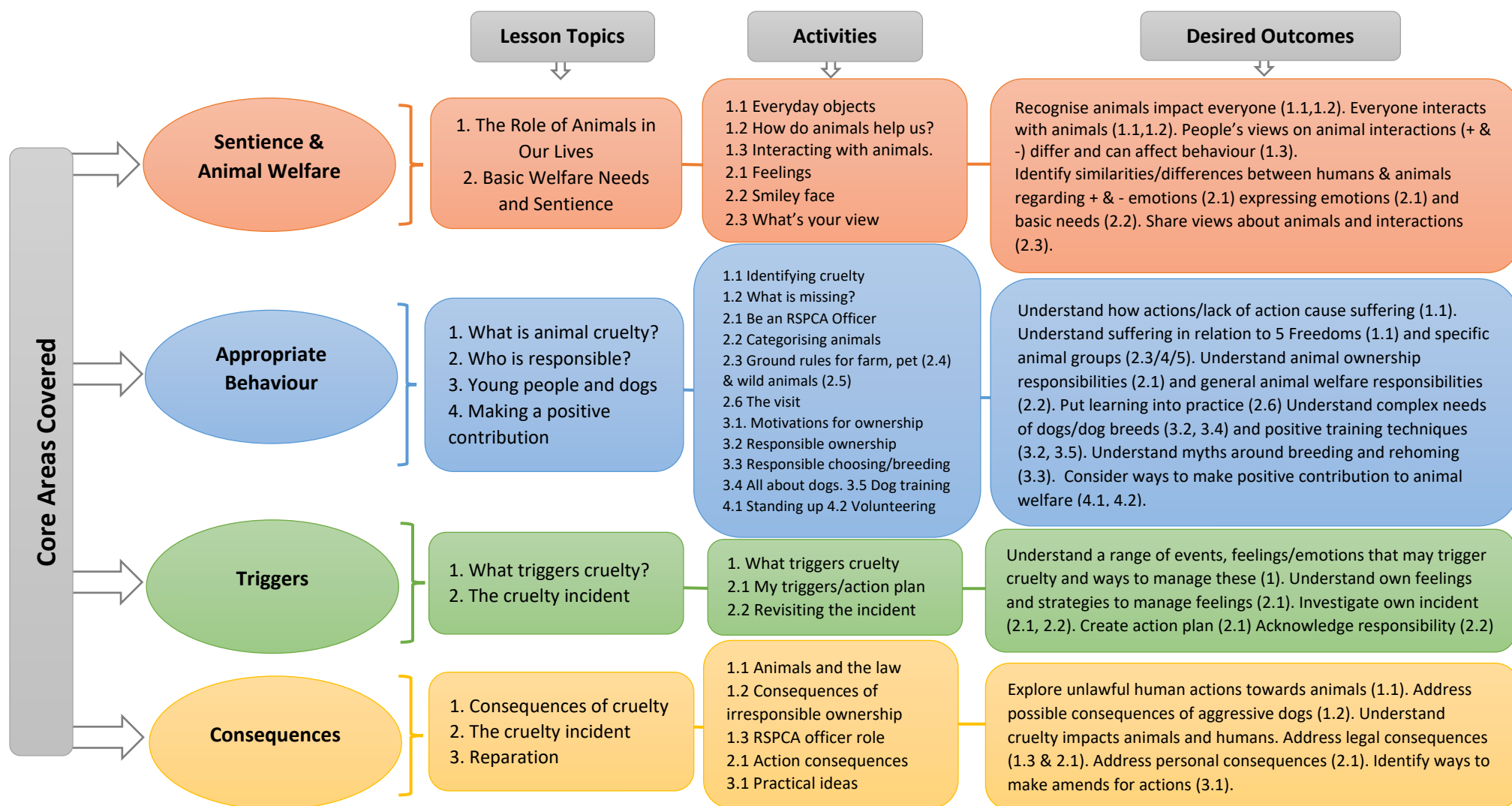

Supplement: Supplementary file 1 [file animals-15-00347-s001.zip › Breaking the Chain Schematic Diagram.pdf]
